# Priorities and Instruments of Local Elderly Care Policies in China: Text Mining and Comparative Analysis

**DOI:** 10.3389/fpubh.2021.647670

**Published:** 2021-07-23

**Authors:** Xiuqi Li, Aoyi Yang, Han Yan

**Affiliations:** ^1^School of Public Policy and Management, University of Chinese Academy of Sciences, Beijing, China; ^2^School of Culture and Communication, The University of Melbourne, Melbourne, VIC, Australia

**Keywords:** elderly care, public health policy, text mining, policy priority, policy instrument

## Abstract

Health care for the elderly is one of the key issues in the field of public health. In the context of global aging, the government's policy framework for elderly care affects the development of local elderly care. The priorities and instruments of the elderly care policy are important windows for understanding the local development planning system. This paper uses a quantitative text analysis method based on text mining to analyze 3,618 provincial policies in China. Considering the pilot demonstration projects for elderly care selected by the Chinese government in recent years, this paper finds that local elderly care policies have a three-phase evolution, and the priorities in each phase are solving the legacy of transition, expanding private sector participation, and realizing the well-being of the elderly. Moreover, mature regions use more environmental policy instruments, and the most effective are financial services, regulatory systems, and strategic guidance. For immature regions, it is necessary to use more core instruments on the premise of using basic instruments so that public policies can serve local development and realize the well-being of the elderly.

## Introduction

In the context of global aging, elderly care is an important issue in the field of public health. Faced with the ever-increasing demand for elderly care and the associated challenges, governments around the world have promulgated a series of policies in the past few decades. Overall, Western developed countries have entered the aging society before countries in other regions (1), and the research on the elderly care policy in these countries is more comprehensive. According to a report of the World Health Organization, China is the country facing the most serious challenge of aging (2). As the world's largest developing country, China has tried to establish a set of institutional arrangements to cope with the increasing number of problems. Focusing on China, many scholars have investigated the policy challenges associated with the elder care system (3), surveyed the effect of the one-child policy on elderly care policy (4), and conducted policy evaluations of new models of elderly care (5). The related studies have mostly focused on the national level, without in-depth study at the local level. However, the level of public service provision at the local level determines the quality of elderly care enjoyed by most citizens. The study of local elderly care policies may help uncover important mechanisms that have been overlooked.

As many previous studies use qualitative case analysis methods, some scholars have innovatively tried to use quantitative methods to carry out research, for example, applying bibliometric analysis to study integrated care policies in China (6). Bibliometric analysis is essentially an automated analysis of political texts (7). Considering the possibility of conceptual confusion, this paper adopts the concept of quantitative text analysis. In recent years, quantitative analysis of political texts has become an important emerging field in public policy research. Studies (8–10) have shown that compared with traditional manual coding, quantitative text analysis has certain advantages in efficiency and accuracy. Traditional qualitative content analysis focuses on combining policy texts in a structured manner, such as exploring policy issues and environments, policy instruments and objectives, and research content in other public policy disciplines. Benefiting from previous theoretical discussion and empirical research, quantitative text analysis has been proven applicable to the study of policy instruments (11), policy topics (12), policy positions (13), and policy diffusion (14). With the help of mature text mining technology, scholars can identify the important information contained in thousands of policy texts.

Identifying the types and discussing the effects of policy instruments are important topics in the context of public policy (15), and policy instruments offer a new perspective for understanding local elderly care policies (16). Policy instruments are the measures used by policy makers to achieve policy goals. The policy instruments supporting elderly care policy are the methods and ways to realize an effective and fair supply of elderly care services. Current studies on policy instruments mainly formulate standards for classifying instruments (17–19), identify the types of policy instruments in specific fields (20), and evaluate specific policy instruments (21). The policies promulgated in a policy area are complex. To understand the crux of the issues, it is necessary to have a comprehensive understanding of the priorities of the issues (22). In the first few decades of the founding of the People's Republic of China, the population structure was relatively young. In 1990, the population over age 65 represented only 5.6% (23) of the total population in China. However, by 2000, the number of elderly people had risen rapidly to 88.21 million (23), and the proportion of people over 65 years of age in the total population had come to exceed 7% (23), such that China officially became an aging society. Since then, elderly care has entered the national policy agenda. The Decision on Strengthening Work on the Elderly promulgated by the Central Committee of the Communist Party of China and the State Council in 2000 can be regarded as the beginning of China's elderly care policy. Subsequently, local governments introduced a series of policies to promote the development of local elderly care. With the promulgation of an important policy in China, the content of related policies will be close to this important policy within a certain period of time. That is, the policy system will show phased characteristics. The phased evolution of policies has become an important window for observing policy development. Based on the existing research and building on its insufficiencies, this paper aims to address the following questions: (1) Is there a phased evolution in China's local elderly care policy? What are the priorities of the policies in each phase? (2) In the current phase, how do local governments use policy instruments to promote the development of local elderly care services, and what effects have they produced? To answer these questions, this paper carries out a specially designed research programme, outlined in the materials and Methods Section.

## Materials and Methods

**Figure 1:**
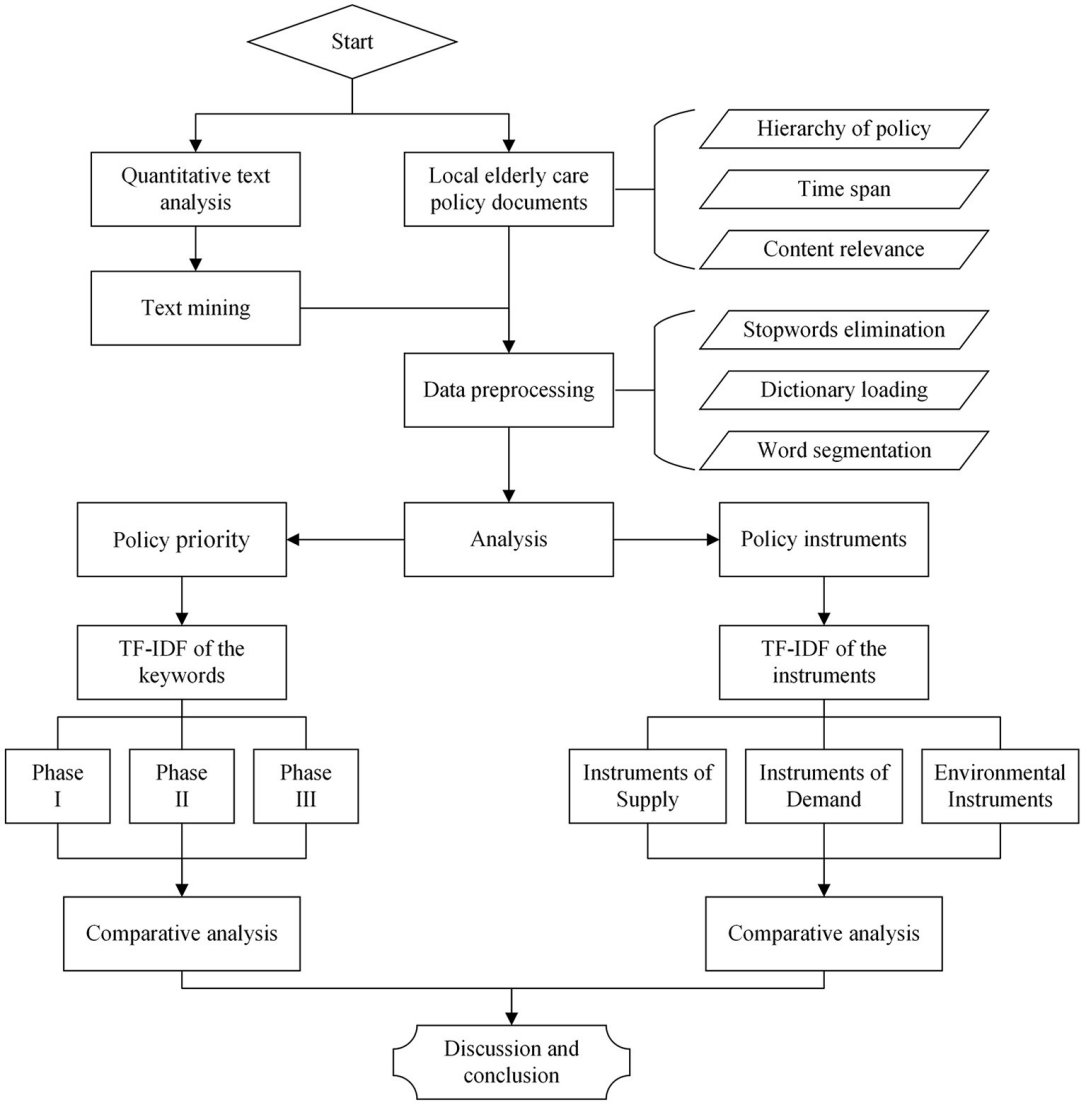
Analysis framework of China's local elderly care policy.

This paper uses quantitative text analysis to study China's local elderly care policies, where policies at the provincial level are regarded as local policies. The document data of local elderly care policies are compiled from the official websites of the provincial governments and the PKUlaw database (see Supplementary Table 1). The analysis framework of this paper is shown in Figure 1.

The logic of the study includes three aspects. First, to understand the phases in the evolution of China's local elderly care policies, this paper analyses the time distribution of policy documents and determines the evolutionary phase of the policy by analyzing important historical events. Second, quantitative text analysis can be used to identify the priorities of policies (7). This study uses text mining technology to mine policy priority terms in different time phases. By sorting the keywords in the text using algorithms, the research team can identify the priorities of China's local elderly care policies in each phase. Third, this study builds on classic theories and studies in China to jointly determine the standard for classifying policy instruments. Then, the type and proportion of policy instruments used by each province are identified by text mining technology. Finally, to understand the effects of the policy instruments, it is necessary to consider the assessment mechanism between the Chinese central government and local governments. The most common assessment mechanism entails the following steps: the central government proposes goals, local governments put them into practice, and the central government assesses and determines pilot demonstration projects. In this study, the research team uses the number of pilot demonstration projects within each region to analyse the effects of policy instruments.

### Acquisition and Processing of Data

**Figure 2:**
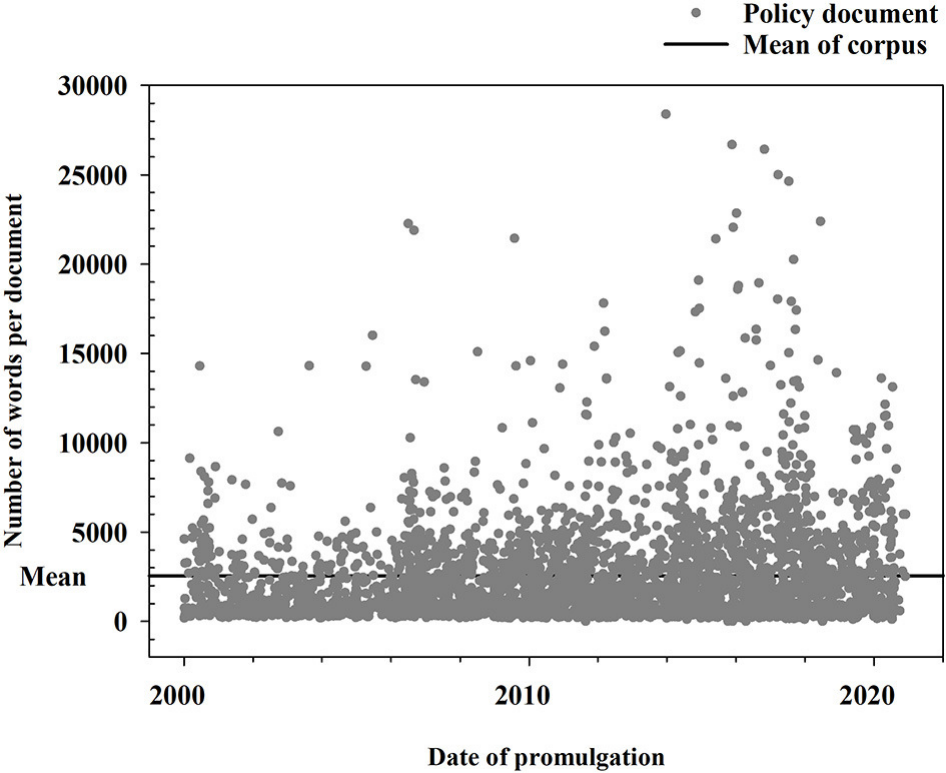
Descriptive statistics of policy corpus.

Specifically, as shown in Figure 1, two members of the research team first searched and sorted out policy documents on the websites and in the database according to the following uniform standards: (1) Policies are promulgated by local governments at the provincial level, and the types include local laws and regulatory documents. (2) The time scale of the policy is from 2000 to 2020. (3) Elderly care as the keyword should be explicitly included in the policy document, not just mentioned. According to this standard, the two research team members obtained a corpus that included 3,618 policy documents after removing duplicate items (see Supplementary Table 2). The word count of the corpus is shown in Figure 2.

The mean number of words per document in the corpus is 2,556, and the total number of words in the corpus exceeds 9.24 million. Then, the whole content is organized into pure text format for text mining.

### Text Mining for Priorities

This paper uses Python 3.7, a mature and reliable programming tool, to mine the priorities and instruments of China's local elderly care policies.

Text mining technology refers to the process of extracting valuable information from textual data (24). Current text mining technology can easily extract keywords, abstracts, or sentiments from unstructured texts (such as news, speeches, policies and other documents). The text mining technology applied in the field of social sciences usually includes data pre-processing, information extraction, sentiment analysis and model construction (25). At present, no study has shown that model construction and sentiment analysis can be effectively applied to Chinese political texts. Therefore, the application of text mining technology in this paper mainly involves information extraction. In general, text mining in Chinese, the information extraction process includes stopword elimination, dictionary loading, word segmentation and the identification of important information (26).

Stopword elimination. There are many meaningless words in a text used for mining, and eliminating stopwords can make the analysis results more reliable (27). This study selected the Chinese stopword list published by the Institute of Computing Technology of the Chinese Academy of Sciences.

Dictionary loading. Loading a custom dictionary can prevent some proper nouns and indivisible concepts from being incorrectly segmented during word segmentation (28). In this study, a suitable custom dictionary was constructed based on the thesaurus of official documents promulgated by the State Council of the People's Republic of China.

Word segmentation. Unlike English words that use spaces as boundaries, the words in Chinese sentences are all coherent. Therefore, word segmentation is essential for the text mining of Chinese texts. This study adopted Jieba (see Supplementary Table 1), a Chinese word segmentation tool based on Python. Then, through the Python program written by the research team, the stopword list and the dictionary list are loaded, and Jieba is run, converting the unstructured continuous policy text into a space-separated corpus.

To understand the main priorities common to many documents, it is necessary to give a certain weight to the keywords. Simple word frequency statistics can easily rank common words instead of keywords in the forefront. In contrast, term frequency-inverse document frequency (TF-IDF) is a better choice (29) that is commonly used as a baseline when comparing keyword extraction algorithms (26). Generally, the frequency of feature items in the document is called the term frequency (TF). For document *d*, the TF value of a certain feature item *t* is calculated as shown in Equation (1):

(1) $$\begin{array}{l} TF_{\left(t|d\right)}=\frac{C_{t|d}}{C_{d}} \end{array}$$

where *C*_*t*|*d*_ represents the number of occurrences of feature item *t* in document *d*, and *C*_*d*_ represents the number of all feature items in document *d*. The TF value considers that feature items that appear more frequently in the same document are more important. However, the obvious defect of TF is that it treats all feature items in the same way, so TF-IDF introduces the inverse document frequency (IDF) to adjust the weight of the TF value. In a corpus *D*, the IDF value of a certain feature item *t* is calculated as shown in Equation (2):

(2) $$\begin{array}{l} IDF_{\left(t|d\right)}=\mathrm{lg}\frac{N}{C_{t|d}+1} \end{array}$$

where *N* represents the number of documents in corpus *D* and adding 1 to the denominator prevents the denominator from being 0. Combining TF and IDF, the calculation of the TF-IDF value is shown in Equation (3):

(3) $$\begin{array}{l} TFIDF_{\left(t|d\right)}=TF_{\left(t|d\right)}\;*\;IDF_{\left(t|d\right)} \end{array}$$

By calculating the TF-IDF for each word in the corpus, the research team can clearly understand the priorities of the local elderly care policy in China.

### Text Mining for Instruments

The most recognized classification standard was proposed by Rothwell and Zegveld. They classify policy instruments into instruments of supply, instruments of demand, and environmental instruments (30). This study adopted this framework and adjusted it with the connotation of China's local elderly care policies. Specifically, opinions concerning elderly care in the tenth to thirteenth 5-Year Plans [the 5-Year Plan for National Economic and Social Development is one of the most important government plans in China, which documents the national strategy during each period (31)] were extracted to form a knowledge base of policy instruments. These instruments were then divided into supply, demand and environmental instruments. Finally, after many discussions with experts in the field of public health and members of the research team, a classification framework of local elderly care policy instruments was determined, as shown in Table 1.

**Table 1:** Classification framework of policy instruments.

**Types of policy instruments**	**Policy instruments**	**Explanation**	**Keyword**
Instruments of supply	Talent training	Talent training policies formulated by the local government to promote the development of the local elderly care industry. For example, providing educational resources, training venues, and qualification certification to promote the overall quality of elderly care service personnel	Talent training; Talent
	Government investment	Financial support provided by the local government for elderly care services. This covers direct grants, conditional incentives and special subsidies	Direct grant; Incentive; Subsidy
	Technological input	Information support provided by the local government for the construction and development of smart communities	Smart community; Information support
	Infrastructure for elderly care	The construction of public nursing homes and elderly care centers led by local governments	Infrastructure
Instruments of demand	Government procurement	The fostering of market-oriented elderly care institutions through direct government procurement of elderly care services	Government procurement
	Service outsourcing	Policies promulgated to innovatively develop local elderly care services, which aim to guide social forces to participate in the construction and operation of local elderly care services	Privatization operation; Operational right
	International cooperation	Local government coordination of international cooperation to regulate the transaction of elderly care services	International cooperation
	Market shaping	The use of media to reduce public opinion barriers and increase market potential	Publicity; Campaign
Environmental instruments	Financial service	Local governments' authorization of financial institutions to raise funds in innovative ways, such as setting up funds, project financing, or unsecured loans to promote the development of the local elderly care industry	Financial service; Funds; Financing
	Tax incentives	Tax breaks and tax benefits offered by the local government to indirectly support the development of the local elderly care service industry	Tax incentives
	Regulatory system	Policies involving handling violations and safety standards promulgated by the local government to improve the standardization level of the elderly care service industry	Regulate
	Strategic guidance	The development goals for elderly care and the guidance of the industry established by the local government	Goal; Programme

The mining of policy instruments and priorities is the same as in the data pre-processing process. The difference is that what is to be extracted is the specific keywords that identify the types of policy instruments. These keywords come from the classification framework.

### The Effects of Instruments

In recent years, the reliance on communities to provide elderly care services with the support of local finance and social capital has become the mainstream form of Chinese policy arrangements. To date, the Ministry of Industry and Information Technology, the Ministry of Civil Affairs and the National Health Commission have jointly announced four batches of the Healthy Elderly care Pilot Demonstration Community (HEPDC). The selection criteria of the HEPDC include the following: (1) The community has a long history of sustainably operating elderly care services. (2) Elderly care services in the community have rich content and strong comprehensive capabilities. (3) Elderly care services in the community have an innovative profit model. In this study, the HEPDCs established in each of the four batches were counted by province. According to the overall quantity distribution, the 31 provincial administrative units (excluding the Special Administrative Regions of Hong Kong and Macau and Taiwan Region) were divided into three types. Then, according to the results of policy instrument mining, statistics regarding the usage of the three types of policy instruments were calculated. Based on this, we analyzed the use and the effects of policy instruments for the development of local elderly care services.

## Result

**Figure 3:**
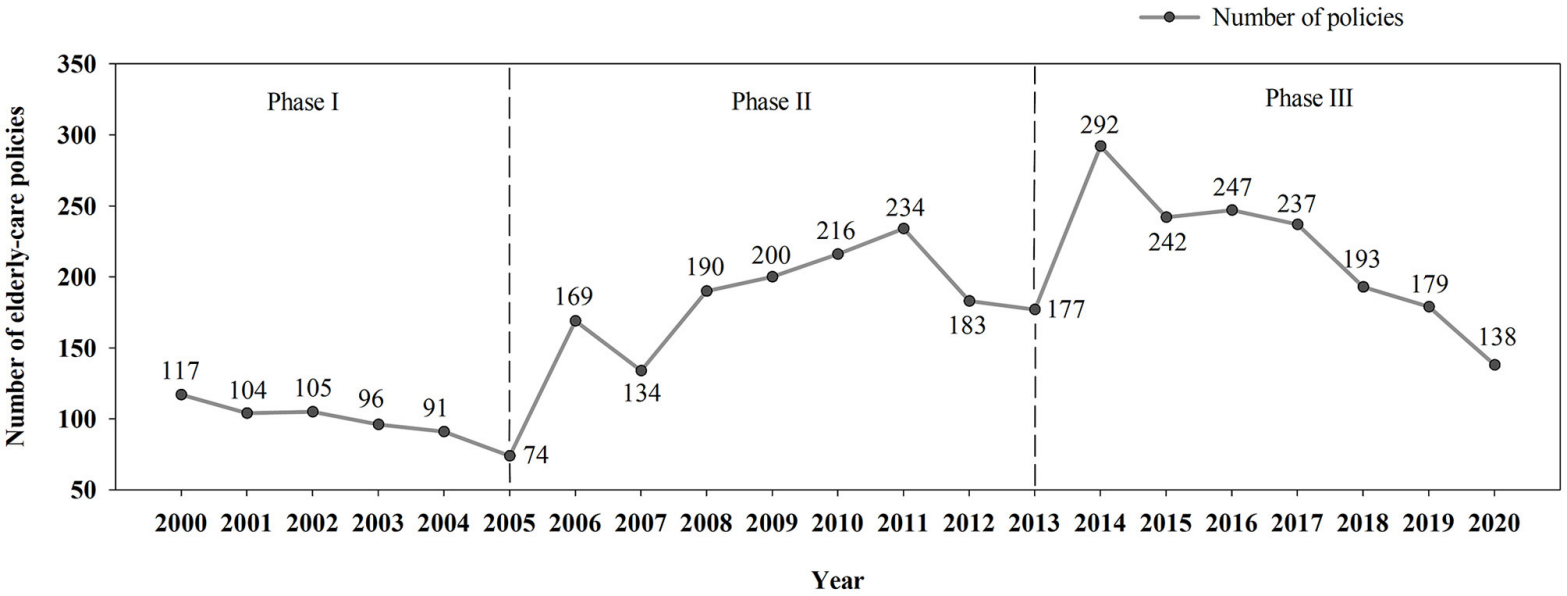
Annual number of the local elderly care policies promulgated in China, 2000–2020.

Figure 3 shows the number of elderly care policies promulgated by local governments in China between 2000 and 2020. In the first few years of the twenty first century, China's elderly care services were still in a transition and exploration phase. Following the call of the central government with the promulgation of the Decision on Strengthening Work on the Elderly in 2000, local governments tried to establish a set of elderly care service systems covering life care, cultural entertainment, and medical care. However, such attempts did not produce explicit results. As a result, the State Council of China promulgated the Circular on Accelerating the Development of the Elderly Care Service Industry in early 2006, which aimed to strengthen the responsibilities of families and communities regarding elderly care services to ease the heavy financial pressure on local governments. In the second decade of the twenty first century, the demand for the quality of elderly care services increased. To further improve the supply capacity of elderly care services, the State Council promulgated the Opinions on Facilitating the Development of the Elderly Care Service Industry at the end of 2013. According to the connotation of the policy and the changing trend in terms of the number of policies, the promulgation time of local elderly care policies in China can be divided into three phases: Phase I lasted from 2000 to 2006; Phase II extended from 2007 to 2013; and Phase III lasted from 2014 to 2020. The following quantitative analysis is based on these three phases.

### The Priorities of Different Phases

After completing the data pre-processing, the research team obtained the corpus of documents from each phase of China's local elderly care policy. Next, the research team calculated the TF-IDF values of all keywords in the corpus of each phase. To visualize the important keywords, a tag cloud was produced based on TF-IDF through the Stylecloud module in Python 3.7 (see Supplementary Table 1).

**Figure 4:**
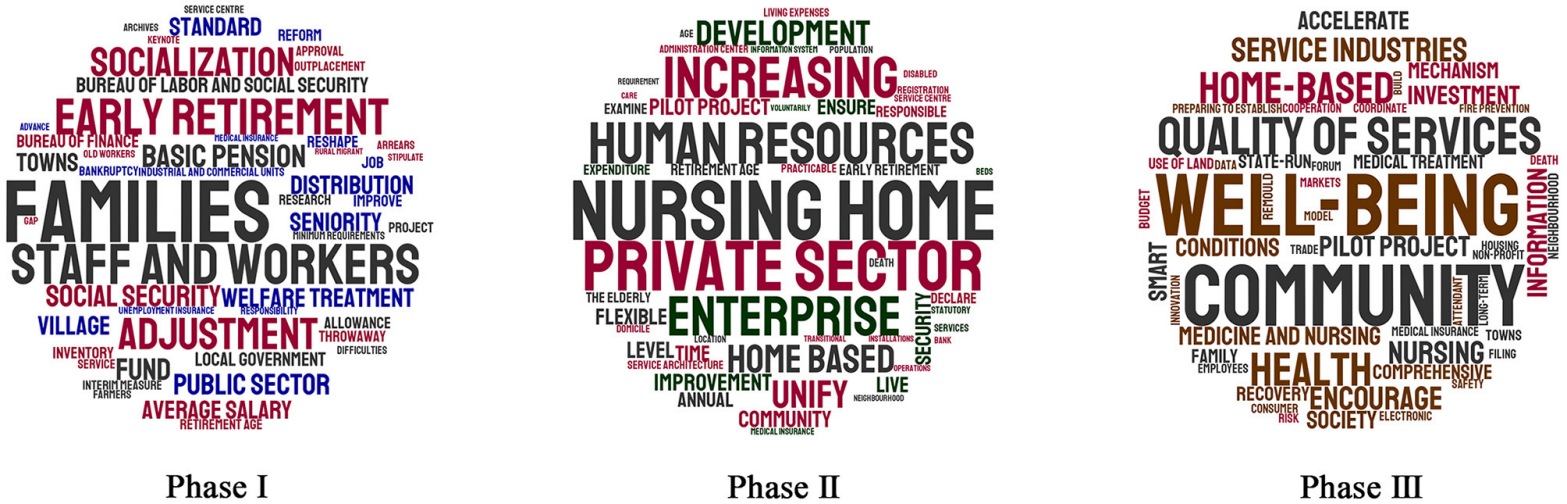
Keywords tag cloud of China's local elderly care policy based on TF-IDF.

As shown in Figure 4, the relatively important keywords in each phase are presented in larger font. In the first phase, families, early retirement, and socialization are the most prominent keywords. This confirms that at the beginning of the new century, local elderly care policies were an accessory embedded in social and fiscal policies. Local elderly care policies had not yet formed an independent policy system. In fact, local governments in China tried to use the power of socialization to solve the problems left over by the transition, and the family was the actual implementer of elderly care. In the second phase, the prominent keywords were nursing homes, the private sector, and enterprises. According to data from the National Bureau of Statistics, the number of local nursing homes increased by more than 15% annually from 2006 to 2013. Among them, more than half of the nursing homes were operated through public-private partnerships (PPPs) and by private actors. In the third phase, well-being, community, and quality of services were prominent keywords. As the average life expectancy continues to increase, people are not satisfied with just food and clothing but have higher demands for quality of life. The Chinese government has come to believe that providing elderly care services through market-based means based on the community can better realize the well-being of the elderly. Therefore, the priorities of China's local elderly care policy are as follows: solving the difficulties of the transition period, exploring the solutions offered by marketization, and gathering strength to achieve the well-being of the elderly.

### Policy Instrument and Its Effect

To make a policy effective, policy makers must understand the scope of the policy instruments they may adopt and clarify the differences among various instruments considered for selection. From the perspective of the policy instrument, policy makers can establish causality between policy goals and outcomes and think about problems based on goals and means, which is an important way to improve the implementation effect.

**Figure 5:**
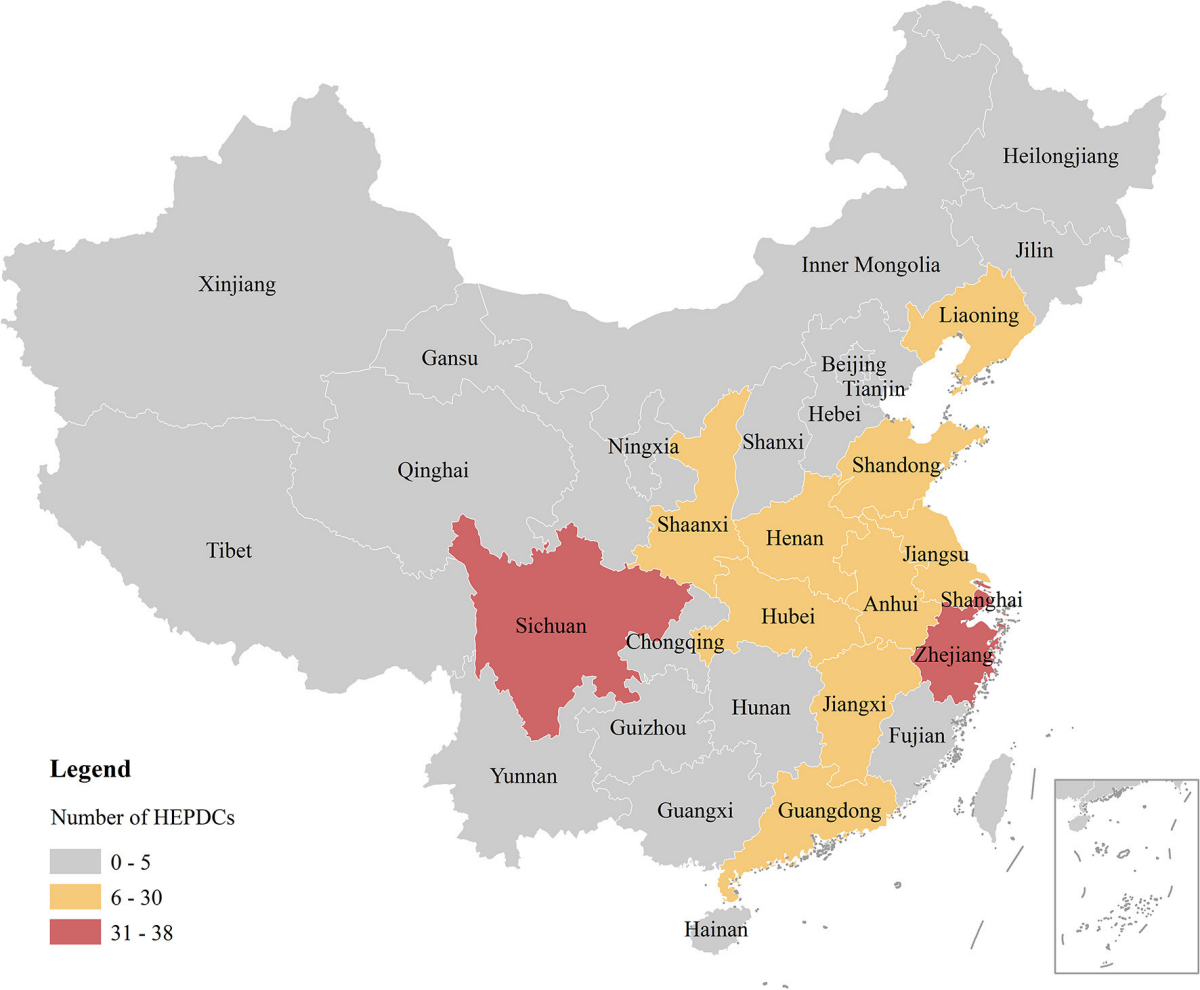
Number of HEPDCs in China by province. Different background colors represent different numbers of projects. HEPDCs are Healthy Elderly care Pilot Demonstration Communities.

To understand the implementation effect of local elderly care policies, the research team considered the statistics for the four batches of the HEPDC. Then, the number of HEPDC projects was calculated by province and visualized, as shown in Figure 5.

Figure 5 shows that each region is divided into three levels according to the number of HEPDCs. Most provinces have no more than 5 HEPDCs. These provinces, marked in gray in the figure, can be called exploratory regions. Some provinces in mid-eastern China have between 6 and 30 HEPDCs. These provinces, marked in yellow, can be called emerging regions. In addition, there are three provinces with more than 30 HEPDCs. These provinces, marked in red, can be called mature regions. Elderly care services differ from other industries in that their profitability is very limited. In most cases, the supply of such public services still depends on the public sector. Government intervention plays a vital role in the transition of local elderly care institutions from the exploration phase to maturity. This kind of intervention is embodied in the use of instruments at the policy level; that is, local governments use diversified policy instruments to improve service quality. According to the classification framework proposed in Table 1, the policy instruments used by local governments include supply instruments, demand instruments and environmental instruments. Supply instruments are basic policy instruments. Direct investment and support from talent and technology can greatly improve supply capabilities in a short period of time and is helpful for improving the level of elderly care services. From the perspective of public economics, the demand and supply of the service market are equally important. The demand instruments used by local governments aim to adjust the heavy burden on the public sector. That is, the government lets the private sector use its intellectual and creative advantages to promote the stability and development of the elderly care service market. Certainly, a good policy environment is also important for the development of local elderly care. Environmental policy instruments, represented by finance, taxation, and regulations, support the achievement of the overall policy goals. Therefore, for regions of different types, are there differences in the use of policy instruments? The research team examined the policy instruments used in each province in a quantitative way, as shown in Table 2.

**Table 2:** The usage of policy instruments in different regions.

**Types of regions**	**Corresponding value range of HEPDCs**	**Number of provinces**	**Type of policy instruments**	**Percentage of the instruments used**
Mature regions	31–38	3	Instruments of supply	28.68%
			Instruments of demand	23.24%
			Environmental instruments	48.09%
Emerging regions	6–30	9	Instruments of supply	50.15%
			Instruments of demand	30.77%
			Environmental instruments	19.08%
Exploratory regions	0–5	19	Instruments of supply	60.98%
			Instruments of demand	21.09%
			Environmental instruments	17.93%

Table 2 shows the number of provinces in different regions and the classification basis between regions. It also shows the types and percentages of policy instruments used in the regions. It must be noted that the percentages in Table 2 are based on regions. That is, (1) Table 1 is used as the standard to calculate the total TF-IDF of specific keywords in the three types of regions; (2) the TF-IDF of these keywords is considered according to the respective policy instruments to obtain various types of the TF-IDF of policy instruments (see Table 3 for details); (3) the three types of regions represent three corpora covering the elderly care policies of the 31 provinces, and the research team normalized the TF-IDF of the keywords in the three corpora. Then, the percentages that reflect the use of policy instruments in different regions were obtained. The other percentages mentioned below were all calculated in this way; the difference is that the normalized objects are different.

**Table 3:** TF-IDF weight of policy instruments in Phase III.

**Type of instruments**	**Instrument**	**Keyword**	**TF-IDF of mature regions**	**TF-IDF of emerging regions**	**TF-IDF of exploratory regions**
Instruments of supply	Talent training	Talent	1.2328	1.0140	2.1639
		Talent training	2.2126	3.1918	6.3711
	Government investment	Direct grant	0.0746	4.1336	11.4434
		Incentive	0.1199	0.7433	2.1120
		Subsidy	0.2495	0.5568	21.3729
	Technological input	Smart community	2.2104	4.8811	2.1363
		Information support	1.8511	3.2854	11.4602
	Infrastructure for elderly care	Infrastructure	0.0715	0.3806	0.9031
Instruments of demand	Government Procurement	Government procurement	0.6008	0.9672	2.1916
	Service outsourcing	Privatization operation	2.2460	3.3949	10.8343
		Operational right	0.5966	2.9071	0.0700
	International cooperation	International cooperation	0.6158	0.4300	1.5392
	Market shaping	Publicity	1.0745	1.4028	4.8977
		Campaign	1.3662	2.0565	0.5182
Environmental instruments	Financial Services	Financial services	1.1246	0.5689	2.6358
		Funds	4.0068	1.0836	2.1463
		Financing	0.3085	0.8244	1.6483
	Tax incentives	Tax incentives	0.4063	0.0859	0.7476
	Regulatory system	Regulation	4.3905	3.2556	7.3970
	Strategic guidance	Goal	1.2960	0.3065	0.6937
		Programme	1.9193	0.7942	1.7722

In this part, the research results will be elaborated from the perspective of the differences in the types of regions and policy instruments.

#### Findings on the Different Regions

**Figure 6:**
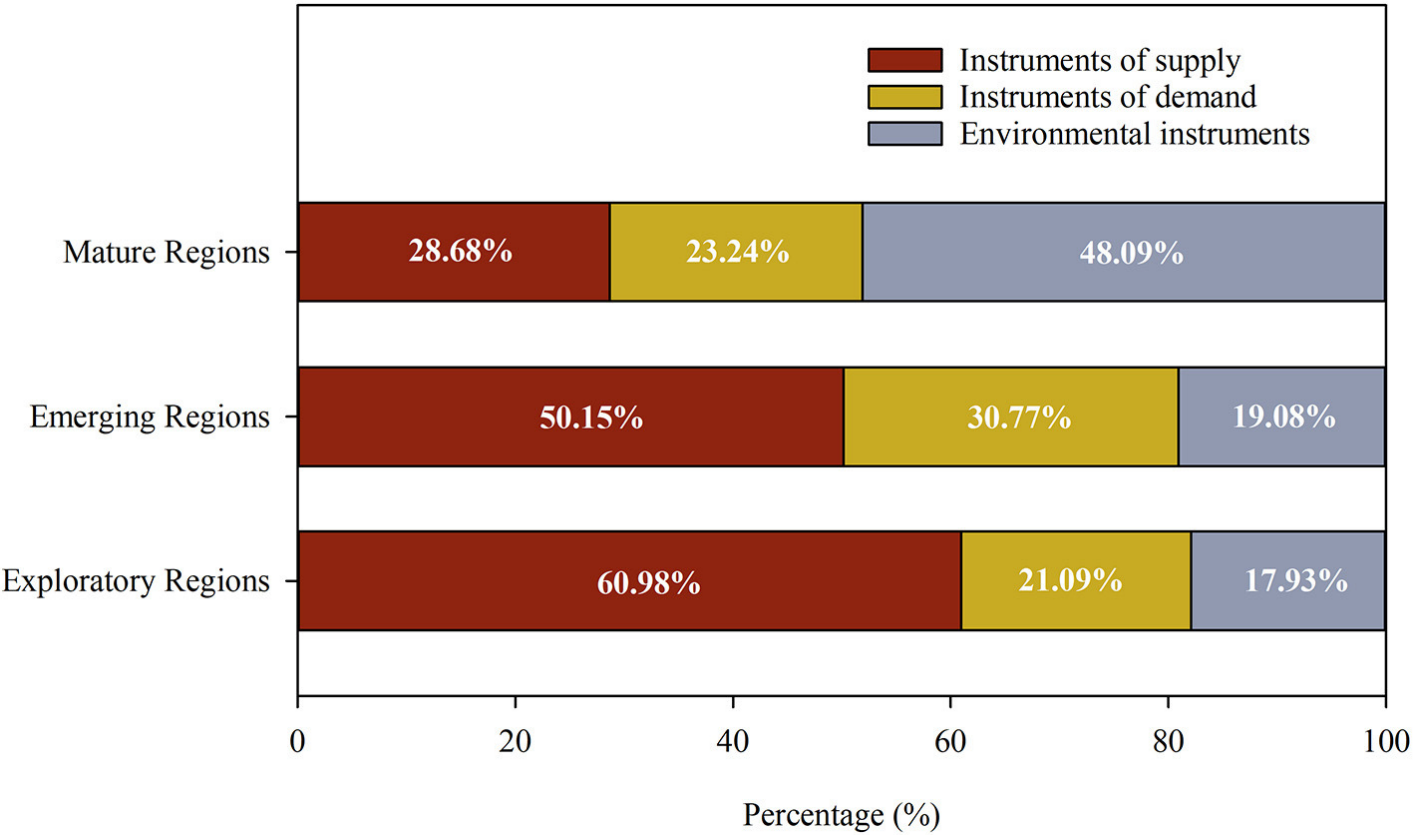
Proportion of instruments used in different regions in China.

The HEPDC represents the Chinese central government's approval regarding the quality of local elderly care services. To understand the usage of policy instruments in different regions at a macro level, the research team normalized the data of the types of policy instruments. As shown in Figure 6, for exploratory and emerging regions with low quality of service, the most used instrument type is the instruments of supply (60.98 and 50.15%). However, for mature regions with a high quality of service, the most used instrument type is environmental instruments (48.09%). In addition, compared with exploratory regions, in emerging regions, the proportion of environmental instruments used increases slightly (from 17.93 to 19.08%), and the more apparently difference is the increase in the proportion of the instruments of demand (from 21.09 to 30.77%).

There are obvious differences in the proportion of instruments used in the three types of regions, which reflects the actual gap in the development of local elderly care. First, the choice of policy tools reflects the governance philosophy of local governments. In essence, the high use of the instruments of supply is a manifestation of exploratory regions' eagerness for quick success. However, in a market economy, this tendency often leads to a mismatch between supply and demand. Instead, placing equal emphasis on demand is a better choice, as shown in emerging regions. Second, all regions have a common policy goal, which is to improve the quality of local elderly care services, and realize the well-being of the elderly. Under this value orientation, the policy effects are even better in mature regions. This also shows that when local governments use a higher proportion of environmental instruments, they can more effectively promote the development of local elderly care.

#### Findings on the Types of Instruments

**Figure 7:**
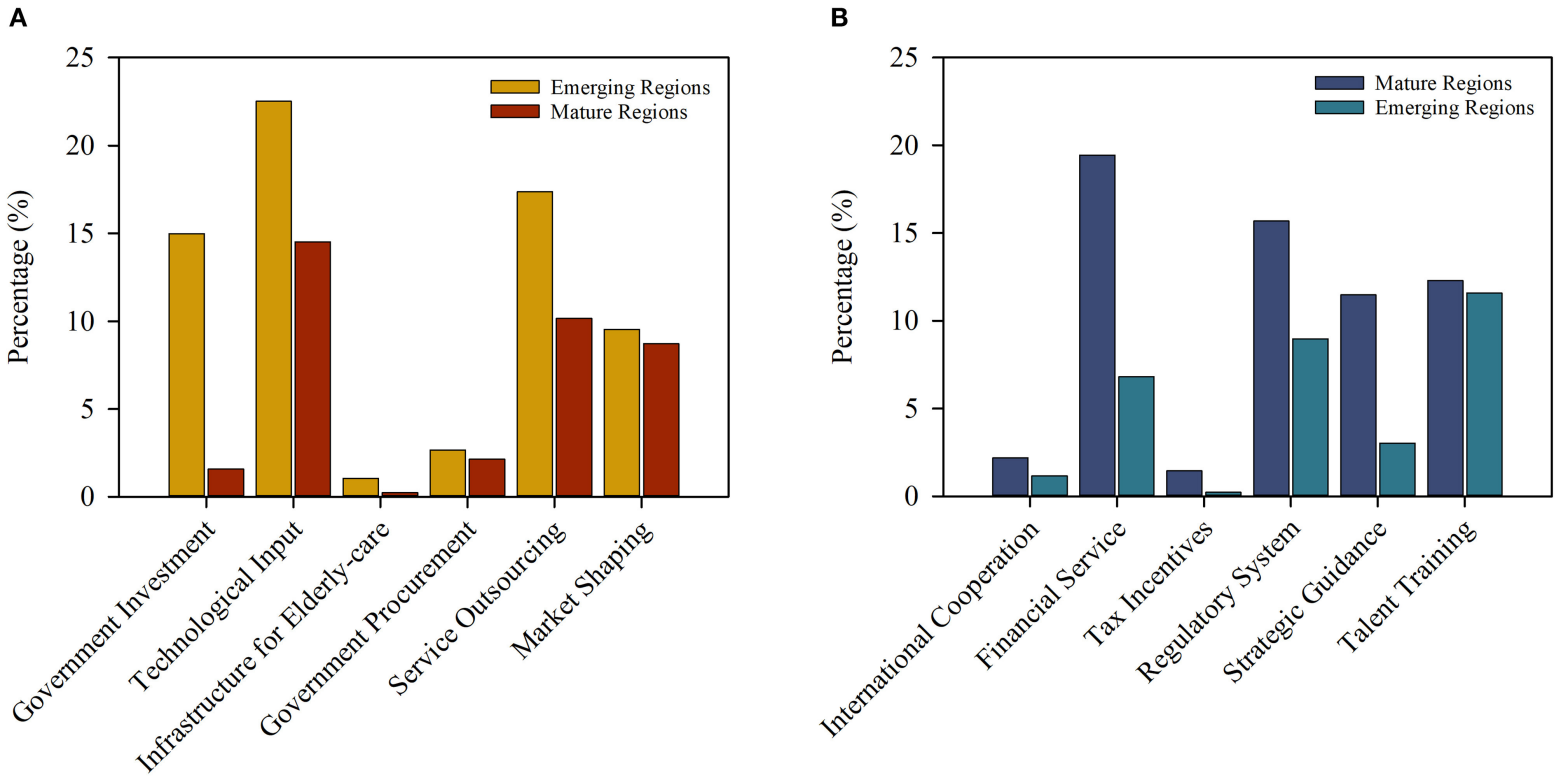
The usage of elderly care policy instruments in mature and emerging regions in China. **(A)** Policy instruments with higher usage rates in emerging regions. **(B)** Policy instruments with higher usage rates in mature regions.

To study the development of local elderly care from the perspective of instruments, it is not enough to understand which type of instruments are more effective. To make practical recommendations, it is important to understand the usage of specific types of instruments. Therefore, the research team normalized the data of the policy instruments and compared the usage of policy instruments in emerging regions and mature regions, as shown in Figure 7.

Figures 7A,B show the elderly care policy instruments with high usage rates in emerging and mature regions, respectively. The figure shows that the policy instruments with similar usage rates between the emerging and mature regions are government procurement with 2.67 and 2.15%, market shaping with 9.54 and 8.72%, international cooperation with 1.19 and 2.20%, tax incentives with 0.24 and 1.45%, talent training with 11.60 and 12.32%, and infrastructure for elderly care with 1.05 and 0.26%. The infrastructure for elderly care refers to basic facilities and equipment for local elderly care, which is necessary for the development of elderly care in exploratory and emerging regions. Talent training is an instrument of supply, and to achieve higher quality of local elderly care, specialized talent covering various disciplines in the field of public health is necessary. The emphasis on talent in various regions also shows that talent training is one of the foundations for the development of local elderly care. Government procurement, market sharing and international cooperation among the instruments of demand are also part of the foundation that supports the local elderly care market. They and the tax incentives among the environmental instruments jointly expand the elderly care service market while adjusting the psychosocial environment in elderly care services on the demand side.

In addition, some policy instruments in emerging regions and mature regions have quite different usage rates, for example, government investment with 14.98 and 1.59%, technological input with 22.52 and 14.52%, and service outsourcing with 17.38 and 10.16%. Government investment and technological input are typical instruments of supply, and they are widely used elderly care policy instruments in emerging and mature regions. Mature regions use fewer supply tools than emerging regions, which is related to the regional differences in whether elderly care services are supplied by the government or the market. This issue is not only related to elderly care services, as it has been part of the reform of local public health and medical systems for decades. Regarding elderly care, mature regions are more inclined to adopt a market-oriented supply mode. This accounts for the efficiency advantages of the private sector and the improvement in supply quality by market-based competition. Of course, what cannot be ignored is that for economically underdeveloped regions, service outsourcing is still an important tool to enhance market vitality. As a quasi-public product, local governments are obliged to equalize the supply of elderly care services through comprehensive measures based on actual local conditions.

Moreover, the usage rates of some policy instruments are greatly higher in mature regions than in emerging regions, for example, financial services with 19.45 and 6.83%, regulatory system with 15.69 and 8.98%, and strategic guidance with 11.49 and 3.04%. Obviously, instead of struggling to meet the responsibilities associated with being an all-powerful government in a context of limited local finances, local governments in mature regions choose to focus on creating a convenient and standardized environment. For small-scale private elderly care institutions, the initial capital is usually not sufficient, and it is difficult to recover costs during the initial stage of operation. In response to this phenomenon, local governments in some mature regions have set up special funds to support start-up elderly care institutions and have given certain credit incentives to institutions operating in a standardized manner. The improvement in the environment of financial services has enabled sporadic small-scale institutions to gradually grow and develop. However, for mature elderly care institutions, a strict regulatory system is more conducive to long-term sustainable development. Finally, public interest is one of the core values of public health. To eliminate the differential treatment and unfairness in elderly care as much as possible, the government's strategic guidance from the macro level is helpful for the realization of health equity.

## Discussion

The above analysis derives the priorities and the policy instruments used in China's local elderly care policies over the past 20 years. The policy priorities are divided into three phases, and the policy instruments are discussed separately according to the development level of elderly care by region. In this part, the study will discuss how local governments of emerging regions and exploratory regions can choose and coordinate policy instruments to promote development to mature regions based on the current priorities of the local elderly care policy.

### Combination of Policy Instruments

Considering the decades of development of the local elderly care services in China, the connotation of related policies tends to be complicated. On the basis of building a basic service system, improving the quality of elderly care and diversifying services has become the focus of recent policies. To achieve the policy goals, local governments in exploratory and emerging regions can learn from the development strategies of mature regions. Improving the usage of policy instruments in exploratory regions and emerging regions will help achieve well-developed local elderly care services in China. First, the basic policy instruments that have laid the foundation for the development of local elderly care must be defined. These basic instruments include infrastructure and talent training to promote supply; government procurement, market sharing and international cooperation to expand demand; and tax incentives to improve the market environment. Second, the core policy instruments also need to be defined. Their usage determines the development level of local elderly care to a certain extent. These core policy instruments include financial services, regulatory systems and strategic guidance. Finally, it is necessary to define some optional policy instruments, such as government investment, technological input and service outsourcing, which can have a good effect under certain conditions.

### Applicability

Elderly care is currently facing many challenges linked to social change and economic inequality, and reasonable development planning of the local government is the fundamental way to face these challenges. This study analyses formal planning from the two dimensions of policy priorities and policy instruments and derives a strategy for guiding the usage of policy instruments in regions where elderly care is underdeveloped. This strategic combination for the development of elderly care not only provides a feasible path for some regions in China but can also be a useful reference for other emerging countries in the world. But this kind of reference should also be based on the actual situation of each country. For example, differences in the financial affordability of countries and the efficiency of the public sector will affect the effectiveness of policy implementation. At the same time, not every emerging country's aging problem is as urgent as China, but some forward-looking discussions will be helpful for these countries to deal with potential problems in the future.

### Limitations

However, this study still has some limitations. First, the research team used the number of HEPDCs in each province to judge the development level of local elderly care. This is only a macro-level assessment, and it does not represent the specific development condition of local elderly care. Second, the research team used quantitative text analysis based on text mining to avoid coarse-grained approaches to policy instrument analysis, such as document coding. However, it is still impossible to avoid the influence of subjective factors of team members when establishing a framework for classifying policy instruments. Even with the help of the expert review, the results of policy instrument analysis remain subject to changes in the classification paradigm. The use of more advanced algorithms or computer-aided programs for analyzing policy instruments may offer greater objectivity and ease for future related research.

## Conclusion

This paper aimed to answer two research questions: (1) Is there a phased evolution in China's local elderly care policy? What are the priorities of policies at each phase? (2) In the current phase, how do local governments use policy instruments to promote the development of local elderly care services, and what effects have they produced? As revealed in the analysis above, China's local elderly care policy has undergone three phases of evolution. Before 2006, the priority of related policies was to solve the problems left over by the transition. From 2006 to 2013, the policy priority was to expand private sector participation in elderly care services, and after 2013, the priority was to provide elderly care services through market-based means based on the community to realize the well-being of the elderly.

In the recent phase, the Chinese government has selected four batches of HEPDCs based on the community elderly care centres' sustainable development, service ability and innovative profitability. Based on the number of HEPDCs, 31 provinces are divided into exploratory regions, emerging regions and mature regions. The most used instruments in mature regions are environmental instruments, while the most used instruments in exploratory and emerging regions are supply instruments. Then, by comparing emerging regions and mature regions, the research team identified that financial services, regulatory systems, and strategic guidance have the most important role in the development of local elderly care. To offer constructive recommendations supporting the development of local elderly care services, this paper divides specific policy instruments into basic instruments, core instruments, and optional instruments.

By providing the above findings and discussion, this paper has made a certain theoretical contribution to the understanding of the relationship between elderly care policy and local development. The analysis of policy instruments and priorities also represents a methodological contribution that may be useful for research in related fields. Considering the limitations and contributions of this paper, future research can enrich the field theoretically and methodologically, for example, by conducting in-depth empirical research based on a certain city or using a large sample for analysis with more mature methods. The analysis of and corresponding adjustment to public policy can turn crises into opportunities. Governments all over the world should be aware of their responsibility to realize the well-being of the elderly through the adjustment of the policy system.

## Data Availability Statement

The original contributions generated for the study are included in the article/Supplementary Material, further inquiries can be directed to the corresponding author/s.

## Author Contributions

XL, HY, and AY: conceptualization. XL: methodology, software, formal analysis, data curation, and writing—original draft preparation. AY: writing—review and editing. XL and HY: visualization. All authors have read and agreed to the published version of the manuscript.

## Conflict of Interest

The authors declare that the research was conducted in the absence of any commercial or financial relationships that could be construed as a potential conflict of interest.

## Publisher's Note

All claims expressed in this article are solely those of the authors and do not necessarily represent those of their affiliated organizations, or those of the publisher, the editors and the reviewers. Any product that may be evaluated in this article, or claim that may be made by its manufacturer, is not guaranteed or endorsed by the publisher.
